# The relationship between obesity and neurocognitive function in Chinese patients with schizophrenia

**DOI:** 10.1186/1471-244X-13-109

**Published:** 2013-04-09

**Authors:** Xiaofeng Guo, Zhanchou Zhang, Qinling Wei, Hailong Lv, Renrong Wu, Jingping Zhao

**Affiliations:** 1 Institute of Mental Health, the Second Xiangya Hospital, Central South University, No. 139 Renmin Mid Road, Changsha, 410011, China; 2 Department of Psychiatry, 3rd Affiliated Hospital of Sun Yat-sen University, Guangzhou, China

**Keywords:** Schizophrenia, Cognitive function, Body mass index, Obesity, Overweight

## Abstract

**Background:**

Studies have reported that up to 60% of individuals with schizophrenia are overweight or obese. This study explored the relationship between obesity and cognitive performance in Chinese patients with schizophrenia.

**Methods:**

Outpatients with schizophrenia aged 18–50 years were recruited from 10 study sites across China. Demographic and clinical information was collected. A neuropsychological battery including tests of attention, processing speed, learning/memory, and executive functioning was used to assess cognitive function, and these 4 individual domains were transformed into a neurocognitive composite z score. In addition, height and weight were measured to calculate body mass index (BMI). Patients were categorized into 4 groups (underweight, normal weight, overweight and obese) based on BMI cutoff values for Asian populations recommended by the World Health Organization.

**Results:**

A total number of 896 patients were enrolled into the study. Fifty-four percent of participants were overweight or obese. A higher BMI was significantly associated with lower scores on the Wechsler Memory Scale-Revised (WMS-R) Visual Reproduction subscale, the Wechsler Adult Intelligence Scale-Revised (WAIS-R) Digit Symbol subscale, and the composite z score (p’s ≤ 0.024). Obese patients with schizophrenia had significantly lower scores than normal weight patients on the Trail Making Test B, the WMS-R Visual Reproduction subscale, the WAIS Digit Symbol subscale, and the composite z score (p’s ≤ 0.004).

**Conclusions:**

Our study suggests that, in addition to its well established risk for various cardiometabolic conditions, obesity is also associated with decreased cognitive function in Chinese patients with schizophrenia. Future studies should explore if weight loss and management can improve cognitive function in obese patients who suffer from schizophrenia.

## Background

Patients with schizophrenia suffer from moderate to severe cognitive impairment especially in the domains of attention, executive functioning, memory, verbal skills, and processing speed impairments [1,2]. Cognitive deficits may represent a core pathophysiological feature of the illness, and these deficits have been found in first episode patients, as well as first-degree relatives of schizophrenia patients [3,4].

Obesity is common in the schizophrenia population [5]. According to recent studies,40-60% of schizophrenia patients are overweight or obese [6-10]. Accumulating evidence indicates that obesity is associated with cognitive dysfunction in non-schizophrenia populations [11-14]. A cross-sectional study of 2,223 healthy workers aged 32 to 62 years showed that body mass index (BMI) was independently associated with cognitive function (word-list learning and the Digit-symbol Substitution Test) [15]. Another study in patients with bipolar disorder suggested that overweight and obesity had a potential negative effect on cognitive function [16]. The cognitive domains most likely adversely affected by excess weight include learning, memory and executive function.

Previous studies in Asian populations have shown that Asians have higher amounts of body fat at lower BMIs than do Western populations, leading to a higher risk for poorer cognitive function in Asian people than in Western people with comparable BMIs [17]. Therefore, there might be differential relationships between obesity as measured by BMI and cognitive function across different racial or ethnic populations.

It has been well documented that overweight or obesity is associated with decreased self-esteem and quality of life, treatment noncompliance, and increased risk for medical comorbidity in schizophrenia [18-20]. However, few previous studies have specifically examined the relationship between overweight or obesity and cognitive function in this patient population. The purpose of this study was to investigate how obesity might be associated with cognitive function in Chinese patients with schizophrenia.

## Methods

### Sample

This study was part of the Early-stage Schizophrenia Outcome Study (ESOS), which involved 10 different sites across China [20]. Study participants were enrolled from psychiatric outpatient clinics. The inclusion criteria were: (1) age range between 18 to 50 years old; (2) diagnosis of schizophrenia or schizophreniform disorder as determined by the Structured Clinical Interview for DSM-IV Axis I Disorders–Clinician Version administered by study investigators or trained staff; (3) duration of illness ≤ 5 years; (4) in stable clinical condition (the Positive and Negative Syndrome Scale [PANSS][21]total scores ≤ 60); (5) on only one oral antipsychotic agent. The exclusion criteria were: (1) a history of neurologic disorders that might affect cognitive function (i.e. head injury, cerebrovascular disease, Parkinson’s disease, Alzheimer’s disease, seizure disorder), (2) serious or unstable medical condition (i.e. poorly controlled diabetes or hypertension, symptomatic coronary artery disease) or BMI ≥ 45 kg/m^2^; (3) current pregnancy or breastfeeding, or history of pregnancy in the past 12 months; (4) substance abuse or dependence in the past 3 months.

The study was performed in accordance with the Declaration of Helsinki and approved by the Institutional Review Board at the *Second Xiangya Hospital, Chongqing Mental Health Center, Psychiatric Hospital of Jiangxi Province, West China Hospital, Mental Hospital of Henan Province, Beijing Anding Hospital, Shanghai Mental Health Center, Guangzhou Brain Hospital, Hunan Brain Hospital, and Nanjing Brain Hospital*. A written informed consent was obtained from each subject, or his or her legal guardians.

### Cognitive assessment

A cognitive battery was administered to all participants. The battery includes the following five cognitive tests:

1) the Wechsler Adult Intelligence Scale-Revised Digit Symbol Test [22]: this is a test of processing speed requiring the examinee to copy symbols that match the numbers 1 through 9 according to a key; the number of symbols completed correctly within 120 seconds was used as the performance measure.

2) Trail Making Test, Part A and B [23]: these tests measure processing speed and executive function, respectively. Part A requires the subject to draw lines connecting numbers in order; Part B requires the subject to alternate between numbers and letters. The time to complete each test was used as the performance measure.

3) The Wechsler Adult Intelligence Scale-Revised Digit Span Test [22]: this test measures attention and working memory and requires the subject to repeat increasingly long strings of digits forward, and then backward. The number of correctly recalled trials in each condition was used as the performance measure.

4) The computerized Wisconsin Card Sorting Test (WCST, 128-card version) [24]: this is a commonly used test for executive function, measuring abstraction and cognitive flexibility; the number of categories completed and the number of perseverative errors were used as the performance measures.

5) The Wechsler Memory Scale-Revised Visual Reproduction Test [25]: this is a visual learning and memory test requiring the subject to copy and recall abstract line drawings; the immediate recall raw score was used as the performance measure.

The eight performance measures were grouped into 4 domains including Processing Speed (Digit Symbol; Trail Making Test, Part A), Attention/Working Memory (Digit Span total), Executive Functioning (Trail Making Test, Part B; WCST perseverative errors and categories completed), and Visual Memory (Visual Reproduction).

All the tests were administered by raters with a masters degree in clinical psychology; an inter-rater reliability was established. The raters were blind to participants’ medical status at the time of assessment.

### Clinical and BMI measurements

Clinical symptoms were assessed using the Positive and Negative Syndrome Scale (PANSS) [21] and the Montgomery and Asberg Depression Scale (MADRS) [26].

Height and weight were measured using the standardized procedures. BMI was calculated based on the formula: weight in kilograms divided by height in meters-squared (kg/m^2^). According to the World Health Organization Standard classification on body mass index for Asians [27], subjects were defined as underweight (< 18.5 kg/m^2^), normal weight (18.5-22.9 kg/m^2^), overweight (23–27.4 kg/m^2^) and obese (≥ 27.5 kg/m^2^).

### Statistical analysis

All analyses were conducted using the Statistical Package for Social Sciences, version 15.0 (SPSS Inc, Chicago, Illinois). A cognitive composite score was derived based on the scores on the five cognitive tests. Raw scores were first converted to standardized z-scores with the mean of 0 and the standard deviation of 1. For domains with more than one test, a domain z-score was created by calculating the mean of the z-scores for the measures comprising the domain, then converting the mean to a z score with a mean of zero and a standard deviation of 1. A cognitive composite score was calculated by creating a z score of the average of the 4 standardized domain scores.

Demographic and clinical characteristics across different BMI groups were compared using analysis of variance (ANOVA) for continuous variables and Chi-square tests for categorical variables. Group comparisons of cognitive function were performed using analysis of covariance (ANCOVA) controlling for gender. If an overall group difference was found to be significant, post hoc comparisons were then evaluated using the Hochberg modification of the Bonferroni correction for multiple comparisons: the adjusted alpha for statistical significance was calculated as α* = α/[k (k-1)/2] =0.05/[4*(4–1)/2] = 0.008 [28]. The correlations between BMI and cognitive function measures were performed using partial correlation tests controlling for both age and gender.

## Results

### Characteristics of the sample

A total of 896 patients completed the study and were included in the data analysis. The prevalence of obesity, overweight, and underweight were 18.1%, 35.6% and 6.4% respectively.

As shown in Table 1, there were no significant differences across different BMI groups (obesity, overweight, normal weight and underweight) on various demographic or clinical characteristics (p’s > 0.05) except for gender (p < 0.001).

**Table 1:** **Demographic and clinical characteristics of the study sample**^**a**^

**Characteristic**	**Underweight (n = 57)**	**Normal weight (n = 358)**	**Overweight (n = 319)**	**Obese (n = 162)**	**F/*χ***^**2**^	**p value**
Age (years)	27.6 ± 8.5	27.1 ± 7.6	27.8 ± 6.6	27.8 ± 7.6	0.738	0.529
Male, N (%)	22(38.6)	162(45.3)	203(63.6)	106(65.4)	36.683	<0.001^b^
Marital Status, N (%)					3.931	0.686
Married	37(64.9)	243(67.9)	197(61.8)	106(65.4)		
Never married	16(28.1)	94(26.3)	103(32.3)	49(30.2)		
Widowed, divorced, or separated	4(7.0)	21(5.9)	19(6.0)	7(4.3)		
Education (years)	12.6 ± 2.8	12.5 ± 3.0	12.4 ± 2.9	12.9 ± 2.8	1.264	0.286
Occupation, N (%)					6.301	0.098
Office worker	20(35.1)	159(44.4)	113(35.4)	66(40.7)		
Manual worker	37(64.9)	199(55.6)	206(64.6)	96(59.3)		
Smoking cigarettes, N (%)	9(15.8)	53(17.4)	61(19.1)	31(19.1)	3.423	0.330
FBS (mg/dl)	88.8 ± 12.7	89.1 ± 12.0	89.3 ± 11.1	92.0 ± 18.8	0.809	0.489
SBP (kPa)	15.1 ± 1.0	14.9 ± 1.2	15.0 ± 1.2	15.2 ± 1.2	2.119	0.096
DBP (kPa)	9.9 ± 1.0	9.8 ± 0.8	9.9 ± 0.8	10.0 ± 0.8	1.996	0.113
DSM-IV diagnosis, N (%)					2.251	0.522
Schizophrenia	52(91.2)	300(83.8)	273(85.6)	137(84.6)		
Schizophreniform disorder	5(8.8)	58(16.2)	46(14.4)	25(15.4)		
Duration of illness (years)	2.2 ± 1.8	2.0 ± 1.7	2.1 ± 1.8	2.4 ± 1.8	1.706	0.164
PANSS total score	47.6 ± 16.5	44.8 ± 14.6	44.8 ± 14.6	46.0 ± 12.9	1.325	0.265
MADRS total score	5.0 ± 5.5	5.2 ± 5.7	5.1 ± 5.5	5.3 ± 5.5	0.106	0.956
Current medication, N (%)					7.063	0.070
Typical antipsychotics	21(31.6)	109(30.4)	71(22.3)	39(24.1)		
Atypical antipsychotics	36(68.4)	249(69.6)	248(77.7)	123(75.9)		
Mean daily doses of medication (clorpromazine quivalent, mg/d)	315.7 ± 107.3	320.8 ± 104.4	322.0 ± 113.0	331.3 ± 115.5	0.447	0.719
Body mass index (kg/m^2^)	16.5 ± 2.4	21.1 ± 1.9	24.9 ± 1.3	29.6 ± 2.4	2203.838	<0.001

^a^Asian BMI categories(kg/m^2^): underweight (< 18.5), normal weight(18.5-22.9), overweight (23–27.4) and obese (≥ 27.5); Data are presented as mean ± SD unless otherwise indicated. Percentages may not sum up to 100 because of rounding.

^b^ underweight vs overweight, p < 0.001; underweight vs obese, p < 0.001; normal weight vs overweight, p < 0.001; normal weight vs obese, p < 0.001;

Abbreviation: *FBS*, fasting blood glucose; *SBP*, systolic blood pressure; *DBP*, diastolic blood pressure; *DSM-IV*, Diagnostic and Statistical Manual of Mental Disorders Fourth Edition; *PANSS*, the Positive and Negative Syndrome Scale; *MADRS*, Montgomery-Asberg Depression Scale.

### Correlations between BMI and cognitive measures

Correlations between BMI and various cognitive measures are showed in Table 2. Partial correlation analysis adjusting for gender demonstrated that a higher BMI was significantly associated with lower scores on the WAIS-R Digit Symbol test, the WMS-R Visual Reproduction test, and the composite z score (p’s ≤ 0.024).

**Table 2:** Partial correlations between body mass index and cognitive function measures controlling for both age and gender

**Characteristic**	**Body mass index**	**p value**
WAIS-R Digit Symbol	−0.076	0.023
Trail Making Test A	0.016	0.641
Trail Making Test B	0.023	0.493
WCST Perseverative Errors	0.005	0.877
WCST Categories Completed	−0.027	0.420
WAIS-R Digit Span	−0.031	0.362
WMS-R Visual Reproduction	−0.131	<0.001
Composite z score	−0.082	0.018

Abbreviation: *WAIS*, Wechsler Adult Intelligence Scale-Revised; *WCST*, Computerized Wisconsin Card Sorting Test; *WMS-R*, Wechsler Memory Scale-Revised; *BMI*, Body mass index.

### Comparison of cognitive measures across different BMI groups

There were significant overall group differences in the Trail Making Test B, the WMS-R Visual Reproduction test, the WAIS Digit Symbol test, and the composite z score (p’s ≤ 0.040; Table 3). Post hoc analysis revealed that the obese group had significantly lower scores on the Trail Making Test B, the WMS-R Visual Reproduction test, the WAIS Digit Symbol test, and the composite z score compared with the normal weight group (p’s ≤ 0.004).

**Table 3:** **Comparisons of cognitive function measures across different BMI groups**^**a**^

**Characteristic**	**Underweight (n = 57)**	**Normal weight (n = 358)**	**Overweight (n = 319)**	**Obese (n = 162)**	**F**	**p value**
WAIS-R Digit Symbol	47.5 ± 15.8	48.3 ± 14.1	46.6 ± 12.8	44.0 ± 13.2	3.922	0.009^b^
Trail Making Test A	56.6 ± 23.7	52.5 ± 24.3	54.9 ± 29.5	55.6 ± 27.3	0.842	0.471
Trail Making Test B	112.3 ± 59.0	106.7 ± 54.6	114.4 ± 61.4	123.7 ± 55.5	2.784	0.040^c^
WCST Perseverative Errors	40.4 ± 25.8	42.7 ± 24.7	42.9 ± 23.7	41.6 ± 26.5	0.241	0.868
WCST Categories Completed	2.7 ± 2.2	2.5 ± 2.1	2.6 ± 2.1	2.8 ± 2.2	0.491	0.688
WAIS-R Digit Span	12.0 ± 3.8	12.5 ± 3.5	12.3 ± 3.1	11.9 ± 3.0	1.606	0.186
WMS-R Visual Reproduction	8.9 ± 2.8	9.3 ± 3.0	8.6 ± 2.9	8.2 ± 3.5	5.921	0.001^d^
Composite z score	0.10 ± 0.09	0.20 ± 0.03	0.10 ± 0.03	0.01 ± 0.05	4.365	0.005^e^

^a^ Asian BMI categories(kg/m^2^): underweight (< 18.5), normal weight(18.5-22.9), overweight (23–27.4) and obese (≥ 27.5); Data are presented as mean ± SD.

^b^normal weight vs obese, P = 0.001; ^c^normal weight vs obese, P = 0.004; ^d^normal weight vs obese, P = 0.001;normal weight vs overweight, P = 0.012 ^e^ normal weight vs obese, P = 0.004;

Abbreviation: *WAIS*, Wechsler Adult Intelligence Scale-Revised; *WCST*, Computerized Wisconsin Card Sorting Test; *WMS-R*, Wechsler Memory Scale-Revised; *BMI*, Body mass index.

### Gender, obesity, and cognitive measures

There was a significant difference in BMI between males and females (24.1 ± 3.7 and 22.8 ± 3.1, respectively, F = 29.872, p < 0.001). However, there were no significant differences in various cognitive measures between the two gender groups (p’s > 0.05; data not shown). Further analysis within each gender group showed that the obese group had significantly lower scores on the WMS-R Visual Reproduction test, the WAIS Digit Symbol test, and the composite z score compared with the normal weight group (p’s ≤ 0.004; data not shown).

### Antipsychotic agents, obesity and cognitive measures

There was a significant difference in BMI between patients on typical antipsychotics and those on atypical antipsychotics (22.9 ± 3.5 and 23.6 ± 3.5, respectively, F = 10.909, p = 0.001), but there were no significant differences in various cognitive measures between those taking typical vs. atypical antipsychotics (p’s >0.05; data not shown).

## Discussion

In this study, the percentage of overweight or obese Chinese patients with schizophrenia was 54%, which is higher than in the general Chinese population (43.1%) [29]. Moreover, we found that obese patients with schizophrenia demonstrated poorer cognitive performance than the normal weight patients. To our knowledge, this is the largest study to assess the relationship between obesity and cognitive function in schizophrenia. The findings of this study suggested that obesity, in addition to being a risk factor for various medical conditions, is associated with decreased cognitive function in patients with schizophrenia.

The exact mechanisms linking obesity and cognitive dysfunction remain unclear. Obesity is associated with vascular changes, impaired insulin regulation, and reduced cardiovascular fitness, which all might contribute to decreased cognitive function [30-32]. In addition, nonvascular mechanisms linking obesity with cognitive impairment have also been suggested [33]. Leptin, an adipocyte-secreted protein related to obesity, may play a role in learning and memory [34]. Interestingly, higher levels of serum leptin appear to protect against cognitive decline in elderly individuals, suggesting leptin resistance as a causal pathway from obesity to cognitive impairment [35]. It is also possible that schizophrenia patients with cognitive impairment are more likely to become obese [12]. If so, much additional work is needed to clarify the association between BMI and worse cognitive performance.

In our study, a higher BMI was found in patients treated with atypical antipsychotics than in those treated with typical agents. However, there was no significant difference in cognitive function between patients on typical vs. atypical antipsychotics. Some atypical antipsychotic agents are associated with significant metabolic side effects; typical antipsychotic agents are more likely to cause other side effects such as extrapyramidal symptoms [36]. This may be the reason why previous studies showed atypical antipsychotic agents are associated with better cognitive function than typical agents in patients with schizophrenia [37].

### Limitations

There are several limitations to this study. First, a more comprehensive cognitive battery might have revealed different results. Second, the participants were not randomly selected and the study excluded patients with serious or unstable medical conditions; as a result, the study sample may not be representative of individuals with schizophrenia in China. Third, given the cross-sectional study design, causal relationships cannot be drawn based on our findings. Fourth, BMI may not be the most appropriate measure to reflect obesity and its negative impact on cardiometabolic disorders and other health conditions [38]. Future studies using more sophisticated techniques, such as dual-energy X-ray absorptiometry, to measure body composition and percentage of fat are needed.

## Conclusion

Our study suggests that, in addition to its well established risk for various cardiometabolic conditions, obesity is also associated with worse cognitive function in Chinese patients with schizophrenia. Future studies should explore if weight loss and management can improve cognitive function in obese patients who suffer from schizophrenia.

## Abbreviations

BMI: Body mass index; WMS-R: The wechsler memory scale-revised; WAIS-R: The wechsler adult intelligence scale-revised; PANSS: Positive and negative syndrome scale; WCST: Wisconsin card sorting test; MADRS: Montgomery and asberg depression scale.

## Competing interests

All authors declare that they have no competing interests.

## Authors’ contributions

GF carried out the study, participated in the analysis and interpretation of data and wrote the manuscript. ZC, WL, LL and WR were involved in carrying out the study and revising the manuscript. ZP conceived of the study and participated in its design and coordination and obtained the funding. All authors contributed to the article and approved the final manuscript.

## Pre-publication history

The pre-publication history for this paper can be accessed here:

http://www.biomedcentral.com/1471-244X/13/109/prepub
